# Computed tomography-based multiple body composition parameters predict outcomes in Crohn’s disease

**DOI:** 10.1186/s13244-021-01083-6

**Published:** 2021-09-25

**Authors:** Ziling Zhou, Ziman Xiong, Qingguo Xie, Peng Xiao, Qingpeng Zhang, Jian Gu, Jing Li, Daoyu Hu, Xuemei Hu, Yaqi Shen, Zhen Li

**Affiliations:** 1 Department of Radiology, Tongji Hospital, Tongji Medical College, Huazhong University of Science and Technology, 1095 Jiefang Avenue, Wuhan, 430030 Hubei China; 2 Biomedical Engineering Department, College of Life Sciences and Technology, Huazhong University of Science and Technology, Wuhan, China; 3 School of Data Science, City University of Hong Kong, Kowloon, Hong Kong China; 4 Department of Anesthesiology, Union Hospital, Tongji Medical College, Huazhong University of Science and Technology, Wuhan, China

**Keywords:** Body composition parameter, Crohn’s disease, Computed tomography

## Abstract

**Background:**

The efficacy of computed tomography-based multiple body composition parameters in assessing disease behavior and prognosis has not been comprehensively evaluated in Crohn’s disease. This study aimed to assess the association of body composition parameters with disease behavior and outcomes in Crohn’s disease and to compare the efficacies of indexes derived from body and lumbar spinal heights in body composition analysis.

**Results:**

One hundred twenty-two patients with confirmed Crohn’s disease diagnoses and abdominal computed tomography scans were retrospectively included in this study. Skeletal muscle, visceral, and subcutaneous fat indexes were calculated by dividing each type of tissue area by height^2^ and lumbar spinal height^2^. Parameters reflecting the distribution of adiposity were also assessed. Principal component analysis was used to deal with parameters with multicollinearity. Patients were grouped according to their disease behavior (inflammatory vs. structuring/penetrating) and outcomes. Adverse outcome included need for intestinal surgery or anti-TNF therapy. Predictors of disease course from multiple parameters were evaluated using multivariate analysis. Indexes derived from body and lumbar spinal heights were strongly correlated (*r*, 0.934–0.995; *p* < 0.001). Low skeletal muscle-related parameters were significantly associated with complicated disease behavior in multivariate analysis (*p* = 0.048). Complicated disease behavior (*p* < 0.001) and adipose tissue parameters-related first principal component (*p* = 0.029) were independent biomarkers for predicting adverse outcomes.

**Conclusions:**

Skeletal muscle and adipose tissue principle component were associated with complicated Crohn’s disease behavior and adverse outcome, respectively. Indexes derived from body and lumbar spinal heights have similar efficacies in body composition analysis.

**Supplementary Information:**

The online version contains supplementary material available at 10.1186/s13244-021-01083-6.

## Key points

- CT-based low skeletal muscle parameters are associated with complicated CD phenotypes.
- Low adipose tissue parameters can predict CD adverse outcomes.
- Lumbar spinal height may be alternative of height in body composition analysis.

## Background

Crohn’s disease (CD) is a chronic, progressive, inflammatory intestinal disorder that is characteristically associated with malnutrition [1, 2]. The increased risk of malnutrition in CD patients may be due to impaired digestion, malabsorption, and inflammatory activity [3]. Malnutrition has a negative impact on the clinical and surgical course of CD and is correlated with worse outcomes in CD patients. The consequence of malnutrition in CD patients is the depletion of muscle mass and changes in fat mass distribution [4]. Mesenteric fat accumulation, as a disease-specific feature of CD, may be correlated with changes in visceral adipose tissue mass or related body composition parameters [5]. Increasing data indicate the correlation of body composition with disease behavior or outcomes in CD patients [6, 7]. Thus, elaborate body composition analysis may serve as a valuable clinical biomarker in CD.

Body mass index (BMI) is a routinely assessed clinical indicator for identifying malnutrition. However, BMI cannot detect skeletal muscle depletion or changes in regional fat distribution [8]. Evaluation of body composition via computed tomography (CT) slice at the level of the third lumbar vertebra allows for accurate prediction of whole-body composition [9] and can thus be used to differentiate and to quantify body tissues in CD patients [10, 11]. CT can be used to directly quantify areas of skeletal muscle, visceral and subcutaneous adipose tissue, mean attenuation of each type of tissue, and lumbar spinal height (ranging from the upper margin of the first lumbar vertebra to the lower margin of the fifth lumbar vertebra) [12].

Identifying prognostic factors in CD is of great clinical significance as complications in CD remain challenging [6, 13]. Imaging biomarker like visceral fat area is used to predict postoperative course [14] or disease activity and outcome [15]. The skeletal muscle and visceral fat areas, normalized by dividing by squared height, are used for further identification of sarcopenia and visceral obesity [16]. The visceral/subcutaneous fat ratio, calculated by dividing the visceral fat area by the subcutaneous fat area, is used as a biomarker of disease behavior in CD [7]. However, few studies simultaneously explored the association of all initially measured parameters and derived indexes with disease behavior and outcome. Moreover, existing results are discordant on the role of certain body composition variables in predicting the post-operative course of CD patients [6, 7, 13–15, 17]. Therefore, it is necessary to comprehensively analyse the role of various original measurement parameters and derived parameters in predicting disease behavior and the outcome of CD patients. Besides, indexes such as the skeletal muscle index and BMI require information on body height, which, inevitably, can be missing in a retrospective study. The use of alternative data (lumbar spinal height), which can be used in place of missing or inaccurate height data, or the adoption of models that predict missing data have not been assessed in the CD population.

The main aim of this study was to assess the association of multiple body composition parameters with disease behavior and outcomes in CD patients. Secondary aim was to compare the efficacy of indexes derived from body height with those derived from lumbar spinal height in body composition analysis.

## Methods

### Patients

The study protocols were approved by the local institutional ethics review board, and informed consent was waived. This study retrospectively included the following population from our institutional database: patients aged > 18 years with a confirmed diagnosis of CD, based on World Gastroenterology Organization Global Guidelines [18], who underwent abdominal CT and were hospitalized between 2012 and 2020. If several admissions existed per patient during the period, only the data from the first admission were used. In all cases, clinical, radiological, endoscopic, histologic findings, and at least 6 months follow-up were used for the confirmed diagnosis of CD. Patients were excluded from the study if: (a) they were diagnosed with inflammatory bowel disease-unclassified; (b) they were diagnosed with confounding comorbidities, such as cancer or severe organ insufficiency; (c) they underwent abdominal surgery in the months before the CT scans; and (d) they had no follow-up data.

### Demographic variables and outcomes

Demographic data, including age, sex, smoking history, height, and weight, were recorded at the time of the abdominal CT scan. The BMI was derived by dividing the weight by the square of the height. Clinical variables of interest included disease duration, perianal disease, a medication used for treatment during admission, disease behavior, and disease location defined according to the Montreal classification [19]. Patients were divided into two groups according to disease behavior: patients with stricturing or penetrating disease were placed into the complicated CD group, and patients with non-stricturing and non-penetrating disease were placed into the inflammatory CD group [7]. Follow-up data within 6 months after the CT, including the need for intestinal surgery, initiation of anti-tumor necrosis factor (TNF) therapy, and escalation of biologic therapy (dosage increase of anti-TNF or change of one anti-TNF agent into another), were collected and used for defining adverse outcomes [16]. We also recorded laboratory markers, including C-reactive protein, erythrocyte sedimentation rate, and serum albumin, within 1 week of the CT scan.

### Quantification of body composition parameters by CT

Digital Imaging and Communications in Medicine images of abdominal CT with coverage of both the abdomen and pelvis were obtained from CD patients for measurements. The lumbar spinal height (ranging from the upper margin of the first lumbar vertebra to the lower margin of the fifth lumbar vertebra) was measured using the sagittal plane in a picture archiving and communication system (Additional file 1: Fig. S1) [12]. A single slice from the abdominal CT per patient at the middle level of the third lumbar vertebra was selected for the segmentation of skeletal muscle, visceral, and subcutaneous adipose tissue. Two radiologists who were blinded to the clinical information of all patients and have extensively trained experience in muscle and adipose tissue area measurements performed the segmentation using ImageJ, version 1.52a (National Institutes of Health, Bethesda, MD, USA). A detailed method of using ImageJ for the quantification of body compositions has been described previously [20]. Semi-automated segmentation was used during these manual courses by using tissue-specific attenuation thresholds such as skeletal muscle (− 29 to 150 HU) and adipose tissue (− 190 to − 30 HU) [21, 22]. An example of segmented each type of tissue was shown in Additional file 1: Fig. S1. The two radiologists also quantified the mean attenuation and cross-sectional area of skeletal muscle, subcutaneous fat, and visceral fat. Further indices [10] were calculated as follow equations:

$${\text{Index(}}i{) } = { }\frac{{{\text{Area}}}}{{{\text{Height}}^{2} }}$$

*i* = skeletal muscle, subcutaneous adipose tissue, and visceral adipose tissue.

And Index(*i*)’ was calculated by dividing lumbar spinal height^2^. The visceral/subcutaneous fat ratio and visceral area divided by the sum of the visceral and subcutaneous areas (visceral/total fat ratio) were used for exploring abdominal fat distributions. To define sarcopenia, skeletal muscle index cutoff values of 28.7 cm^2^/m^2^ for females and 49.9 cm^2^/m^2^ for males were applied as previously proposed [23]. Cut-off values of skeletal muscle index_spinal_ for defining sarcopenia were further assessed by using receiver operating characteristic curve analysis.

We assessed inter-observer agreement of measurements of the area and mean attenuation of each type of tissue and lumbar spinal height using intraclass correlation coefficients.

### Statistical analyses

Descriptive parameters are expressed as the mean ± standard deviation. Cases missing data on height and BMI (missing due to missing height) were imputed using multiple imputations, correlating the height by the age, sex, and areas of skeletal muscle, and visceral and subcutaneous adipose tissue. The mean value of the five imputed height data was used for subsequent analysis or derivation of body composition parameters. The *χ*^2^ test or fisher’s exact test was used to compare categorical variables. An independent *t*-test or the Mann–Whitney test was used for quantitative parameters. Pearson’s test was used for correlation analysis between variables, and coefficients were presented in the correlation temperature map. Principal component analysis using the “princomp” function in R software was used to derive new variables that can retain most of the information of the original body composition parameters (with Pearson correlation coefficient > 0.7). The principal component with an eigen value > 1 was enrolled for further univariate and multivariate analyses [24].

Variables associated with complicated behavior and with *p* < 0.2 in univariate logistic regression analysis were chosen in a backward stepwise multivariate logistic regression model [16]. Odds ratios (ORs) with 95% confidence intervals (CIs) were summarized in the final model. Kaplan–Meier curves for continuous variables below the median (vs. above the median) for the overall population were compared using log-rank test. Cox regression analysis was performed to estimate possible risk factors of adverse outcomes in CD. All statistical significance levels were set at *p* < 0.05. All analyses were performed using IBM SPSS Statistics for Windows, version 23.0 (IBM Corp., Armonk, N.Y., USA) and R software version 3.6.0 (R Project for Statistical Computing).

## Results

### Study population

**Fig. 1:**
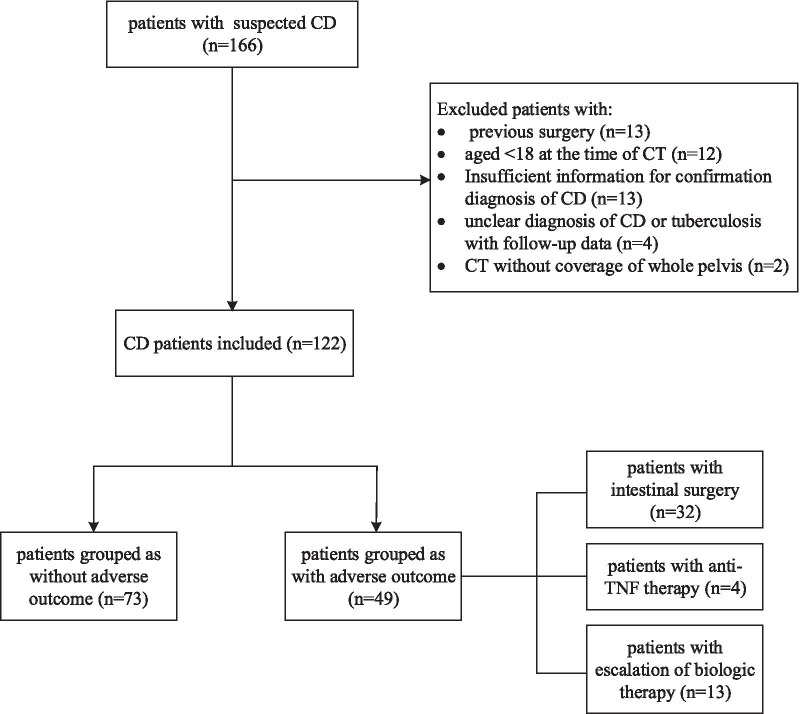
Flowchart of study population. *CD* Crohn’s disease, *CT* computed tomography

A total of 122 CD patients with abdominal CT scans were enrolled (Fig. 1). The mean age of the patient cohort at hospitalization was 32.5 years (range 18–70 years). Of the study population, 22.1% were women, and 12.3% were current smokers. The mean disease duration was 2.19 years (range 1 month–16 years). There were 32 patients who presented with perianal diseases. The prevalence of sarcopenia in the entire study population was 84/122 (68.85%). Most of the patients were in the inflammatory group (*n* = 68; 55.74%), and fewer were in the complicated group (*n* = 54; 44.26%). The demographic characteristic and clinical information of the cohort are shown in Table 1. The two groups were well-matched regarding age, sex, duration of disease, smoking status, disease location, and laboratory indexes. Patients in the inflammatory group had a higher BMI than those in the complicated group (inflammatory group, 19.19 ± 2.82; complicated group, 17.88 ± 2.66; *p* = 0.034). Treatments during hospitalization, including nutritional supplementation and mesalazine, were significantly more frequent in patients in the inflammatory group.

**Table 1:** Baseline characteristics of the study population, stratified by disease behavior

	All	Complicated	Inflammatory	*p* value
*Demographic data*	*n* = 122	*n* = 54	*n* = 68	
Age (years)	32.5 ± 12.04	33.85 ± 12.70	30.79 ± 11.41	0.165
Sex				0.181
Male	95	39	56	
Female	27	15	12	
Current smoker				0.062
Yes	15	10	5	
No	107	44	63	
Location^a^				0.263
L1	49	26	23	
L2	27	11	16	
L3	46	17	29	
Perianal disease				0.630
Yes	32	13	19	
No	90	41	49	
Duration of disease (years)	2.19 ± 2.93	2.33 ± 2.90	2.08 ± 2.97	0.653
C-reactive protein (mg/L)^b^	43.03 ± 41.41 (*n* = 107)	47.02 ± 48.08 (*n* = 44)	40.25 ± 36.19 (*n* = 63)	0.408
Serum albumin (g/L)^b^	35.13 ± 6.67 (*n* = 120)	34.66 ± 6.78 (*n* = 54)	35.52 ± 6.57 (*n* = 66)	0.481
Erythrocyte sedimentation rate (mm/h)^b^	26.59 ± 22.75 (*n* = 111)	27.67 ± 23.82 (*n* = 46)	25.82 ± 22.12 (*n* = 65)	0.674
BMI (kg/m^2^)	18.49 ± 2.80	17.88 ± 2.66	19.19 ± 2.82	**0.034**
Sacorpenia				0.604
Yes	84	39	45	
No	38	15	23	
*Baseline medications*				
Diagnostic anti-tuberculosis therapy	30	8	22	**0.025**
Nutritional supplement	106	51	55	**0.028**
Anti-TNF	20	11	9	0.290
Corticosteroids	30	9	21	0.070
Immunomodulator	16	5	11	0.261
Mesalazine	58	20	38	**0.038**
*Follow-up outcomes*				
Adverse outcome	*n* = 49	*n* = 31	*n* = 18	0.775
Surgery	32	20	12	
Start of anti-TNF therapy	4	2	2	
Escalation of biologic therapy	13	9	4	

Continuous data are expressed as means ± SDs; *p* values lower than 0.05 are presented in bold

*BMI* body mass index, *TNF* tumor necrosis factor

^a^L1: ileal, L2: colonic, and L3: ileocolonic (Montreal classification)

^b^Part of patients with missing data on C-reactive protein, serum albumin, and erythrocyte sedimentation rate

During follow-up, a total of 49 patients were classified as having adverse outcomes: 32 underwent intestinal surgery, 4 initiated anti-TNF therapy, and 13 needed escalation of biologic therapy (Table 1).

### Body composition and nutritional parameters

**Fig. 2:**
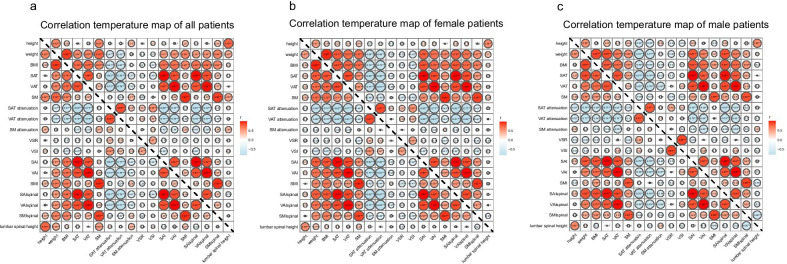
Correlation temperature map describing correlations between multiple body composition parameters in the 122 study patients. Correlation temperature map of body composition parameters in all patients (**a**), female patients (**b**), and male patients (**c**). *BMI* body mass index, *SM* skeletal muscle, *SAT* subcutaneous adipose tissue, *VAT* visceral adipose tissue, *SMI* SM index, *SAI* SAT index, *VAI* VAT index, *SMI*_*spinal*_ SM area/lumbar spinal height^2^, *SAI*_*spinal*_ SAT area/lumbar spinal height^2^, *VAI*_*spinal*_ VAT area/lumbar spinal height^2^, *VSR* area of VAT/area of SAT, *VSI* area of VAT/(area of VAT + area of SAT)

At the time of CT, there were 40 patients with missing data on height and thus BMI. After imputation, the mean BMI was 18.49 ± 2.80 kg/m^2^, and there was no significant difference between males (18.70 ± 2.81 kg/m^2^) and females (17.74 ± 2.67 kg/m^2^, *p* = 0.197). Interobserver agreement was strong for lumbar spinal height and area of each type of tissue (intraclass correlation coefficients ranging from 0.726 to 0.997, *p* < 0.001). In the correlation temperature map, the correlation between height and lumbar spinal height was moderate (*r* = 0.756, *p* < 0.001, Fig. 2) using original and imputed data from the 122 CD patients and was moderate (*r* = 0.780, *p* < 0.001, Additional file 1: Fig. S2) using original data from the 82 CD patients. Indexes derived from body height and lumbar spinal height were strongly correlated (*r*, 0.934–0.995; *p* < 0.001). Correlations among areas of skeletal muscle, and indexes of skeletal muscle were strong (*r*, 0.888–0.940; *p* < 0.001) with a slightly more pronounced correlation in male patients (Fig. 2). Mean attenuations of subcutaneous adipose tissue and visceral adipose tissue were moderately correlated (*r* = 0.853; *p* < 0.001). Variables including BMI, area of subcutaneous and visceral fat, and indexes of subcutaneous and visceral adipose tissue, were moderately correlated (*r*, 0.689–0.995; *p* < 0.001; Fig. 2). The first skeletal muscle principal component (reflecting skeletal muscle status and were named skeletal muscle principle component; Additional file 1: Fig. S3) was extracted from the original skeletal muscle related variables including area and attenuation of skeletal muscle, and indexes of skeletal muscle using principal component analysis. In addition, adipose tissue related variables, including area of subcutaneous and visceral adipose tissue, attenuation of subcutaneous and visceral adipose tissue, and indexes of subcutaneous and visceral adipose tissue, were weights averaged to obtain the first adipose tissue principle component (reflecting adipose tissue status and were named adipose tissue principal component; Additional file 1: Fig. S3). Equations showing the association between the two extracted first principal components and their original variables (which were *z*-score normalized) with weights were as follows:

1. Skeletal muscle principal component = 0.562 * area of skeletal muscle + 0.564 * skeletal muscle index + 0.549 * skeletal muscle index_spinal_ + 0.254 * attenuation of skeletal muscle;
2. Adipose tissue principal component = 0.364 * area of subcutaneous adipose tissue + 0.358 * area of visceral adipose tissue + 0.363 * subcutaneous adipose tissue index + 0.359 * visceral adipose tissue index + 0.361 * subcutaneous adipose tissue index_spinal_ + 0.359 * visceral adipose tissue index_spinal_ − 0.330 * attenuation of subcutaneous adipose tissue − 0.332 * attenuation of visceral adipose tissue

**Fig. 3:**
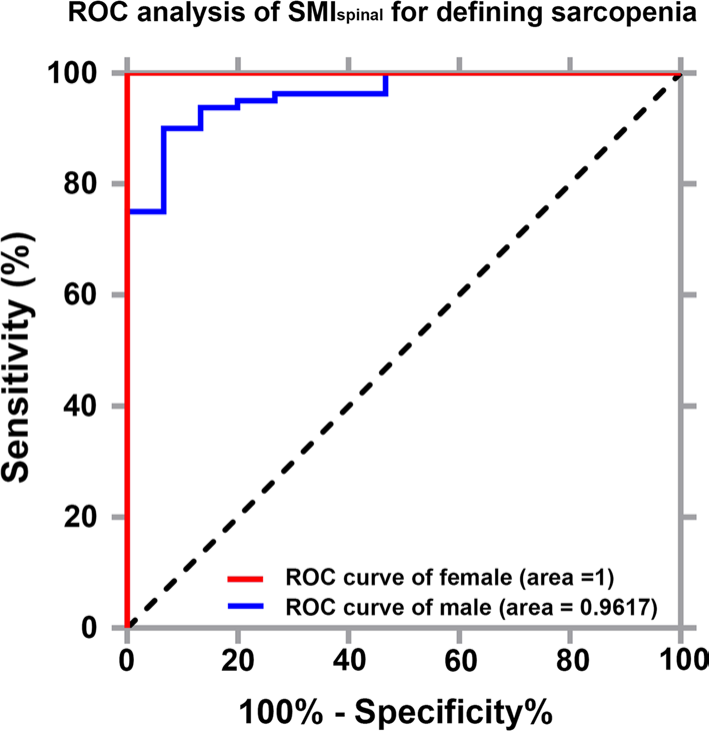
Receiver operating characteristic curve analysis of SMI_spinal_ index cut-off values for defining sarcopenia. *SMI*_*spinal*_ skeletal muscle area/lumbar spinal height^2^

Receiver operating characteristic analysis showed that the skeletal muscle index_spinal_ cut-off value for defining sarcopenia was 4.707 × 10^3^ cm^2^/m^2^ (sensitivity, 90.00%; specificity, 93.33%; *p* < 0.001) and 2.597 × 10^3^ cm^2^/m^2^ (sensitivity, 100%; specificity, 100%, *p* < 0.001) in male and female patients, respectively (Fig. 3).

### Relationship between body composition parameters and disease behavior in CD patients

**Fig. 4:**
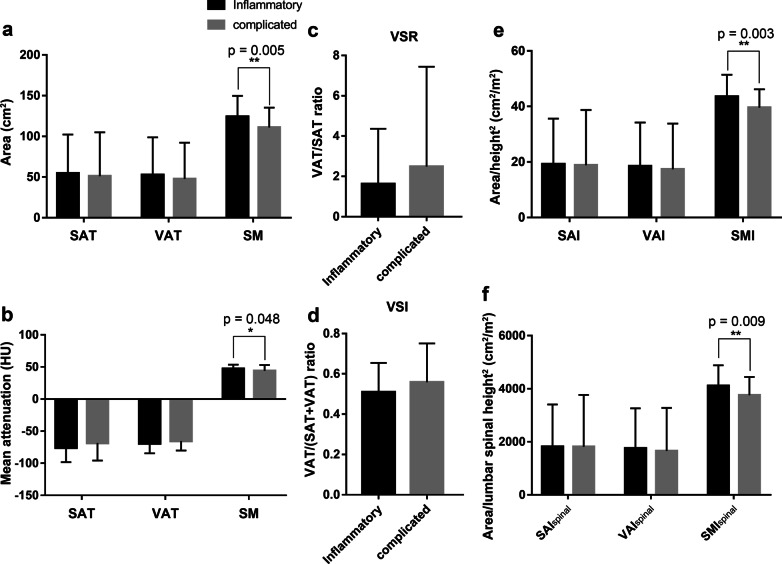
Body composition in complicated and inflammatory CD. **a** Area of SAT, VAT, and skeletal muscle in complicated and inflammatory CD; **b** mean attenuation of SAT, VAT, and skeletal muscle in complicated and inflammatory CD; **c** VSR in complicated and inflammatory CD; **d** VSI in complicated and inflammatory CD; **e** Value of SAI, VAI, and SMI in complicated and inflammatory CD; **f** Value of SAI_spinal_, VAI_spinal_, and SMI_spinal_ in complicated and inflammatory CD. The data are presented as means ± SD, *p* values by two-tailed unpaired *t* tests. *SAT* subcutaneous adipose tissue, *VAT* visceral adipose tissue, *VSR* area of visceral fat/area of subcutaneous fat, *VSI* area of visceral fat/(area of visceral fat + area of subcutaneous fat), *SAI* subcutaneous adiposity index, *VAI* visceral adiposity index, *SMI* skeletal muscle index, *SAI*_*spinal*_ subcutaneous adiposity area/lumbar spinal height^2^, *VAI*_*spinal*_ visceral adiposity area/lumbar spinal height^2^, *SMI*_*spinal*_ skeletal muscle area/lumbar spinal height^2^

When grouped by disease behavior, patients in the inflammatory group had higher values of BMI, area of skeletal muscle and adipose tissue, mean attenuation of skeletal muscle, and derived parameters (indexes of skeletal muscle, subcutaneous fat, and visceral fat) than patients in the complicated group (Fig. 4). Patients in the inflammatory group had lower values of visceral/subcutaneous fat ratio, visceral/total fat ratio, subcutaneous adipose tissue, and mean attenuation of visceral adipose tissue than those in the complicated group. Values of skeletal muscle principal component reached statistical differences between the two groups in univariate analysis (Table 2). In multivariate analysis, skeletal muscle principal component (OR, 728; 95% CI 0.531–0.997; *p* = 0.048) with lower values remained associated with more complicated disease behavior (Table 2). Skeletal muscle principal component was derived based on the area and attenuation of the skeletal muscle and skeletal muscle indexes. Thus, lower areas and attenuation of the skeletal muscle and skeletal muscle indexes were indicators of more complicated disease behavior.

**Table 2:** Univariate and multivariate analyses of factors associated with disease behavior

Variable	Univariate OR (95% CI), *p* value	Multivariate OR (95% CI), *p* value
Age	1.021 (0.991, 1.053), 0.170	1.025 (0.992, 1.059), 0.132
Female sex	1.795 (0.758, 4.251), 0.184	0.898 (0.237, 3.405), 0.874
Skeletal muscle principal component	0.714 (0.568, 0.897), **0.004**	0.728 (0.531, 0.997), **0.048**
Adipose tissue principal component	0.958 (0.835, 1.098), 0.536	–
Visceral/subcutaneous fat ratio	1.069 (0.970, 1.178), 0.180	1.037 (0.932, 1.154), 0.503
Visceral/total fat ratio	5.774 (0.657, 50.743), 0.114	2.418 (0.085, 68.948), 0.605

*CI* confidence interval, *OR* odds ratio, visceral/subcutaneous fat ratio, area of visceral fat/area of subcutaneous fat, *p* values lower than 0.05 is presented in bold

### Body composition parameters associated with adverse outcomes in CD patients

**Fig. 5:**
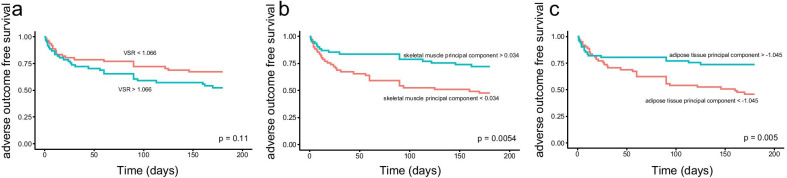
Adverse outcome free survival. Kaplan–Meier curves depicting adverse outcome free survival for the overall population stratified by VSR (**a**), skeletal muscle principal component (**b**), and adipose tissue principal component (**c**). *VSR* area of visceral fat/area of subcutaneous fat

Some body composition parameters (area and mean attenuation of subcutaneous and visceral adipose tissue, skeletal muscle index, visceral/subcutaneous fat ratio, visceral/total fat ratio, and subcutaneous adipose tissue indexes) of patients without adverse outcomes, measured at baseline CT, were significantly different from those of patients with adverse outcomes (Table 3). Low BMI (*p* = 0.012) was more frequent in patients with adverse outcomes. Kaplan–Meier curves for patients stratified using the median value of visceral/subcutaneous fat ratio, skeletal muscle principal component, and adipose tissue principal component showed that adverse outcome rate was lower for patients with lower visceral/subcutaneous fat ratio, higher skeletal muscle, and higher adipose tissue principal component (Fig. 5). Univariate analysis showed that complicated disease behavior (stricturing and penetrating), low adipose tissue principal component, and high visceral/subcutaneous fat ratio were associated with more frequent adverse outcomes (Table 4). Low adipose tissue principal component indicated high attenuation of subcutaneous and visceral adipose tissue, low area and indexes of subcutaneous adipose tissue, and low area and indexes of visceral adipose tissue. Thus, high attenuation of adipose tissue and low adipose-related body composition parameters were risk factors of adverse outcomes. In multivariate analysis, complicated disease behavior, older age, and low adipose tissue principal component remained related with adverse outcomes in CD patients (Table 4).

**Table 3:** Comparison of body composition between patients with different outcomes

	With adverse outcome	Without adverse outcome	*p* value
*Demographic data*	*n* = 49	*n* = 73	
Age (years)	33.51 ± 14.54	31.23 ± 10.04	0.308
Sex			0.412
Male	40	55	
Female	9	18	
Current smoker			0.583
Yes	7	8	
No	42	65	
Location^a^			0.258
L1	16	33	
L2	14	13	
L3	19	27	
Disease behavior			**0.001**
Inflammatory	18	50	
Complicated	31	23	
Duration of disease (years)	1.70 ± 2.51	2.52 ± 3.15	0.128
*Body composition and nutritional parameters*			
C-reactive protein (mg/L)^b^	48.51 ± 39.70 (*n* = 42)	39.49 ± 42.41 (*n* = 65)	0.274
Serum albumin (g/L)^b^	34.53 ± 6.52 (*n* = 49)	35.55 ± 6.80 (*n* = 71)	0.413
Erythrocyte sedimentation rate (mm/h)^b^	30.93 ± 24.53 (*n* = 42)	23.94 ± 21.35 (*n* = 69)	0.117
BMI (kg/m^2^)	17.61 ± 2.41	18.85 ± 2.78	**0.012**
SAT area (cm^2^)	39.18 ± 45.61	62.37 ± 51.44	**0.012**
VAT area (cm^2^)	40.08 ± 42.98	57.54 ± 45.59	**0.036**
SM area (cm^2^)	112.74 ± 22.56	122.03 ± 27.26	0.051
SAT attenuation (HU)	− 65.31 ± 24.83	− 77.27 ± 24.03	**0.009**
VAT attenuation (HU)	− 64.24 ± 13.89	− 69.72 ± 15.45	**0.048**
SM attenuation (HU)	45.19 ± 8.54	46.06 ± 7.36	0.546
SAI (cm^2^/m^2^)	14.35 ± 17.11	22.14 ± 17.99	**0.018**
VAI (cm^2^/m^2^)	14.56 ± 16.06	20.27 ± 15.67	0.053
SMI (cm^2^/m^2^)	40.15 ± 6.81	42.93 ± 7.86	**0.046**
SAI_spinal_ (× 10^3^ cm^2^/m^2^)	1.395 ± 1.739	2.097 ± 1.719	**0.030**
VAI_spinal_ (× 10^3^ cm^2^/m^2^)	1.477 ± 1.648	1.967 ± 1.744	0.076
SMI_spinal_ (× 10^3^ cm^2^/m^2^)	3.810 ± 7.004	4.052 ± 7.745	0.081
Visceral/subcutaneous fat ratio	8.06 ± 25.98	1.22 ± 0.99	**0.026**
Visceral/total fat ratio	0.58 ± 0.20	0.50 ± 0.14	**0.010**
Sarcopenia			0.271
Yes	37	47	
No	12	26	

Continuous data are expressed as means ± SDs, *p* values lower than 0.05 are presented in bold

*BMI* body mass index

^a^L1: ileal, L2: colonic, and L3: ileocolonic (Montreal classification)

^b^Part of patients with missing data on C-reactive protein, serum albumin, and erythrocyte sedimentation rate; *SM* skeletal muscle, *SAT* subcutaneous adipose tissue, *VAT* visceral adipose tissue, *SMI* SM index, *SAI* SAT index, *VAI* VAT index, *SMI*_*spinal*_ SM area/lumbar spinal height^2^, *SAI*_*spinal*_ SAT area/lumbar spinal height^2^, *VAI*_*spinal*_ VAT area/lumbar spinal height^2^

**Table 4:** Univariate and stepwise multivariate Cox proportional hazard regression analysis for prediction of short time outcome in CD

Variable	Univariate analysis		Multivariate analysis	
	HR, 95% CI	*p* value	HR, 95% CI	*p* value
Age	1.021 (0.997, 1.045)	0.095	1.040 (1.010, 1.070)	**0.008**
Sex (female)	0.783 (0.380, 1.613)	0.506	0.575 (0.246, 1.346)	0.202
Visceral/subcutaneous fat ratio	1.012 (1.002, 1.021)	**0.016**	1.001 (0.991, 1.012)	0.818
Skeletal muscle principal component	0.852 (0.725, 1.001)	0.051	–	–
Adipose tissue principal component	0.873 (0.769, 0.991)	**0.035**	0.872 (0.772, 0.986)	**0.029**
Perianal disease (yes)	1.595 (0.885, 2.873)	0.120	1.841 (0.989, 3.427)	0.054
Smoking status (yes)	1.151 (0.517, 2.563)	0.731	0.625 (0.268, 1.456)	0.276
Disease behavior (complicated)	2.865 (1.600, 5.131)	**< 0.001**	3.244 (1.749, 6.012)	**< 0.001**

*CD* Crohn’s disease, *HR* hazard ratio, *CI* confidence interval; visceral/subcutaneous fat ratio, area of visceral fat/area of subcutaneous fat, *p* values lower than 0.05 are presented in bold

## Discussion

This study comprehensively compared the correlation of various body composition parameters with disease behavior and outcomes in CD patients. It has been shown that multiple body composition parameters derived from body and lumbar spinal heights have the same efficacies in predicting CD course. Patients with complicated disease behavior tend to have lower skeletal muscle mass and indexes. Complicated disease behavior and high attenuation and low area indexes of adipose were predictive factors of adverse outcomes in CD patients.

This study showed that CD patients with lower skeletal muscle mass and related parameters tend to have more complicated disease courses. Lower skeletal muscle index has been noted in studies regarding cancer and CD patients [25–27]. Meanwhile, sarcopenia, defined by a skeletal muscle index lower than the cut-off value, has been shown to be a predictor of poorer prognosis in cancer and CD patients [16, 27]. CD patients are more likely to have reduced skeletal mass than ulcerative colitis patients and healthy controls [25, 28]. Sarcopenia tends to be increased in patients with unfavorable outcomes [16, 29, 30]. Malnutrition, chronic inflammation, and immobility are potential causes of sarcopenia in CD patients. These factors are also predictors of a severer CD course [17, 30]. However, when there is a strong correlation among body composition parameters, separately analyzing the relationship between a parameter and the disease course may magnify the predictive ability of the parameter for the CD course. Therefore, principal component analysis was used to eliminate the joint part influence of each variate in differentiating CD disease behavior. The study found that all skeletal muscle-related parameters, including the area of skeletal muscle and indexes of skeletal muscle, rather than adiposity were indicators of complicated disease behavior. Patients with complicated disease behavior may benefit from intervention aiming at improving skeletal muscle-related parameters.

Visceral fat tissue accumulation is supposed to be obvious in CD patients with stricturing and penetrating phenotypes [15]. Given that cytokines produced by visceral fat in CD may consequently lead to mesenteric fat hypertrophy [31], it is reasonable to believe that patients with a higher proportion of fat in the visceral area have more complicated disease behavior. Values (area and mean attenuation) and indexes of visceral fat cannot identify patients with complicated disease behavior after multivariate analysis in present study. As Erhayiem et al. reported visceral/subcutaneous fat ratio to be an independent factor of complicated CD [7]. Kedia et al. showed that a visceral/subcutaneous fat ratio > 0.63 was a feature of CD that could be used as a differential diagnosis from tuberculosis [32], and the mean visceral/subcutaneous fat ratio in our CD population was > 1. Besides, visceral/subcutaneous fat ratio was an important factor for indicating outcome. Moreover, penetrating and stricturing phenotypes were also risk factors of adverse outcomes, confirming the results of Bamba et al. [10]. The results of our study add to evidence regarding the role of body composition in Crohn’s disease. Based on the features of all initially measured body composition parameters and derived indexes in baseline CT scans of CD patients, timely medical intervention can be taken to prevent worse prognosis.

Quantification of body composition from CT scans appears to be more accurate than the BMI and most appropriate for assessing multiple body composition parameters in CD patients. The area of skeletal muscle and skeletal muscle indexes was capable of identifying a complicated course of CD in our study. However, body height captured in medical records may be patient self-reported and influenced by multiple factors such as development and aging. On the other hand, lumbar spinal height measurements can be performed as long as an abdominal CT scan with coverage of the abdomen, and pelvis is available. Lumbar spinal height was not used in body composition analysis previously, and there are no uniform standard criteria for measuring it. Therefore, we used the upper and lower margins of the first and fifth lumbar vertebrae, respectively, in an initial attempt. The missing height data appeared to be random in our study; therefore, we adopted a multi-imputation model to compute the missing height data and subsequent indexes. Our results revealed a high correlation between indexes derived from squared height and those derived from lumbar spinal height. Measuring spinal height and area of each type of tissue from CT concurrently may enhance the reliability of all kinds of derived indexes. The findings of our study revealed that comprehensive skeletal muscle parameters were biomarkers of a complicated CD course, and attenuation and indexes of adipose were significant body composition parameters in predicting the outcome in CD. Indexes derived using different method have the same efficacies in body composition analysis which helps extend the nutritional status assessment in CD patients. Further studies are needed to confirm whether to use single or multiple body composition parameters in identifying a complicated disease course of CD.

There are a few limitations to our study. First, we defined exclusion criteria which excluded some patients with abdominal surgery before the CT scans to eliminate influence of surgery on fat quantification. Thus, our conclusions were specific at the population of the center who meet the inclusion and exclusion criteria, whereas selection bias may be unavoidable in our single center. Second, we adopted same 6 months period as Thiberge et al. [6] follow-up data to evaluate the association between body composition parameters and short-term outcome. Attention should be paid that our prognostic-related results were not fully applicable for reflecting the outcome of patients with long-term disease course. Further research with extension of follow-up time is needed to find out the relationship between body composition parameter and long-term outcome. Third, there were missing data in some patients, such as C-reactive protein, erythrocyte sedimentation rate, albumin, and height. The missing data were supposed to have no influence in the results of our study. Finally, disease behavior was classified according to Montreal classification at baseline, and subsequent change in disease behavior during follow-up was not considered.

## Conclusions

In conclusion, skeletal muscle mass-related parameters are lower in patients with complicated disease behavior. Complicated disease behavior, high attenuation, and low area indexes of adipose tissue are common in CD patients with adverse outcomes. Indexes derived from lumbar spinal height have the same efficacy with indexes derived from body height in evaluating the correlation of multiple body composition parameters with CD disease behavior and outcomes.

## Supplementary Information

**Additional file 1.** **Fig. S1.** Measurement of body composition using cross-sectional computed tomography slice. **Fig. S2.** Correlation temperature map describing correlations between multiple body composition parameters in 82 study patients with no missing height data. **Fig. S3.** Screen plot and biplots of components in the two principal component analysis models of the body composition parameters.

## Data Availability

The dataset used or analyzed during the current study are available from the corresponding author on reasonable request.

## Abbreviations

BMI: Body mass index
CD: Crohn’s disease
CT: Computed tomography
TNF: Tumor necrosis factor
