# Supplementary material for: Computed tomography-based multiple body composition parameters predict outcomes in Crohn’s disease
Source: Insights Imaging. 2021 Sep 25;12:135. doi: 10.1186/s13244-021-01083-6 (PMC8464641; doi:10.1186/s13244-021-01083-6)
Supplement: Supplementary file 1 — Additional file 1. Fig. S1. Measurement of body composition using cross-sectional computed tomography slice. Fig. S2. Correlation temperature map describing correlations between multiple body composition parameters in 82 study patients with no missing height data. Fig. S3. Screen plot and biplots of components in the two principal component analysis models of the body composition parameters. [file 13244_2021_1083_MOESM1_ESM.docx]

**Computed tomography-based multiple body composition parameters predict outcomes in Crohn’s disease**

**Additional file 1**

**
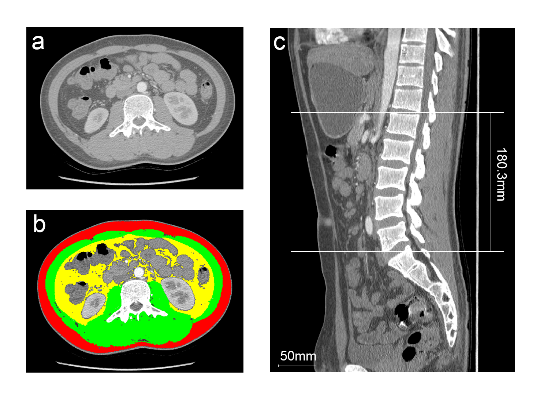
**

**Fig. S1. Measurement of body composition using cross-sectional computed tomography slice.** a, CT slice at the middle level of the third lumbar of one CD patient; b, segmentation result of skeletal muscle (green), subcutaneous adipose tissue (red), and visceral adipose tissue (yellow). c, sagittal slice showing way of measuring lumbar spinal height in the same patient.

**
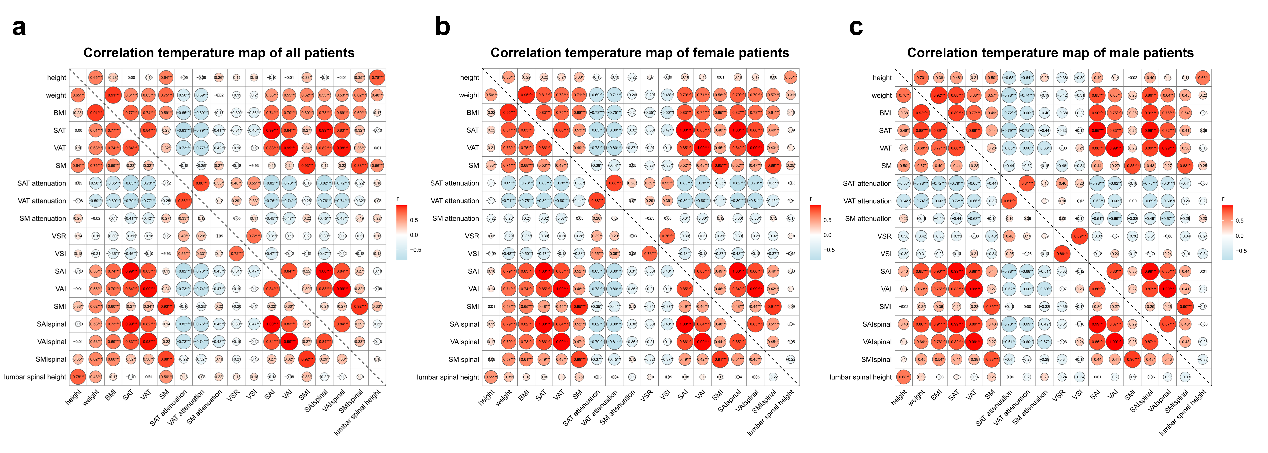
**

**Fig. S2. Correlation temperature map describing correlations between multiple body composition parameters in 82 study patients with no missing height data.** Correlation temperature map of body composition parameters in all patients (a), female patients (b), and male patients (c). BMI, body mass index; SM, skeletal muscle; SAT, subcutaneous adipose tissue; VAT, visceral adipose tissue; SMI, SM index; SAI, SAT index; VAI, VAT index; SMI_spinal_, SM area/lumbar spinal height^2^; SAI_spinal_, SAT area/lumbar spinal height^2^; VAI_spinal_, VAT area/lumbar spinal height^2^; VSR, area of VAT/area of SAT; VSI, area of VAT/(area of VAT+ area of SAT).


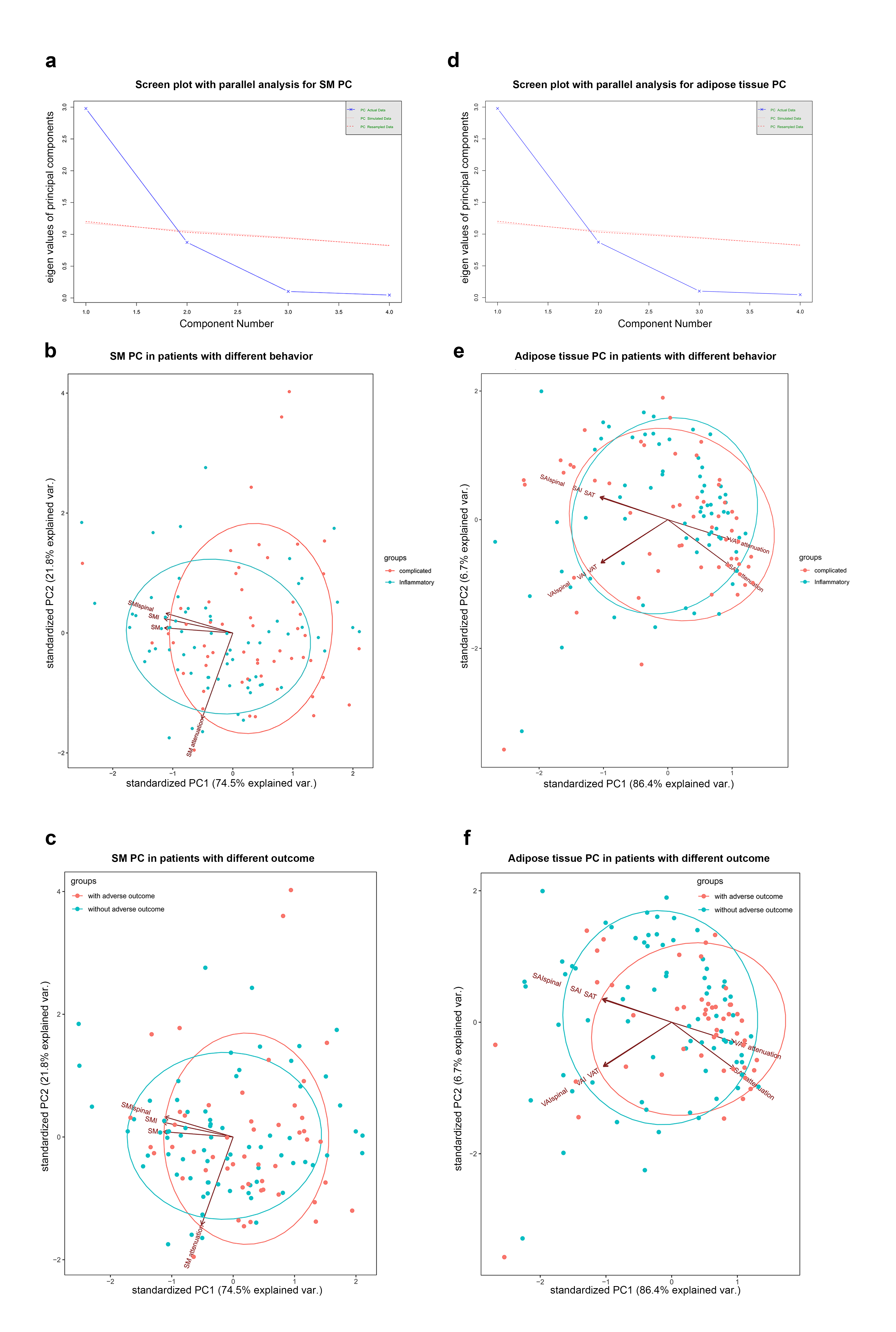


**Fig. S3. Screen plot and biplots of components in the two principal component analysis models of the body composition parameters.** Screen plot showed that eigen value was larger than one only in skeletal muscle principal component and adipose tissue principal component (a and d). Skeletal muscle principal component (b) and adipose tissue principal component (e) in patients placed into inflammatory CD group and complicated CD group; Skeletal muscle principal component (c) and adipose tissue principal component (f) in patients with different outcomes. BMI, body mass index; SM, skeletal muscle; SAT, subcutaneous adipose tissue; VAT, visceral adipose tissue; SMI, SM index; SAI, SAT index; VAI, VAT index; SMI_spinal_, SM area/lumbar spinal height^2^; SAI_spinal_, SAT area/lumbar spinal height^2^; VAI_spinal_, VAT area/lumbar spinal height^2^; VSR, area of VAT/area of SAT; VSI, area of VAT/(area of VAT + area of SAT); PC, principal component.
